# A Case of Delayed Interval Delivery with a Successful Hospital Move

**DOI:** 10.1155/2015/802097

**Published:** 2015-08-30

**Authors:** Toshifumi Yodoshi, Elizabeth Tipton, Christopher A. Rouse

**Affiliations:** ^1^Department of Pediatrics, United States Naval Hospital Okinawa, Camp Foster, Futenma, Ginowan, Okinawa, Japan; ^2^Department of Obstetrics and Gynecology, United States Naval Hospital Okinawa, Camp Foster, Futenma, Ginowan, Okinawa, Japan; ^3^Department of Neonatology, United States Naval Hospital Okinawa, Camp Foster, Futenma, Ginowan, Okinawa, Japan

## Abstract

This report is the first case of delayed interval twin delivery in which the first infant and mother survived without major morbidity following transport to another facility. In addition, this case is only the second report of asynchronous delivery in which both twins survived and neither suffered any major morbidity. A 30-year-old G_5_P_1031_ African American female with a diamniotic/dichorionic twin pregnancy presented to U.S. Naval Hospital Okinawa, Japan, at 22 + 5 weeks due to vaginal bleeding. At 23 + 2 weeks, Twin A was born secondary to advanced cervical dilation. Twin A's birth weight was 650 g with APGAR scores of 6 (1 min) and 7 (5 min). Following delivery of Twin A, Placenta A was left in utero with high ligation of the umbilical cord. Due to a scheduled hospital move, the mother and Twin A were transported to the new facility at Camp Foster. Three weeks later, Twin B was delivered at 26 + 4 weeks. Twin B's birth weight was 930 g with APGAR scores of 3 (1 min) and 7 (5 min). Both twins were discharged without IVH, PVL, ROP, or CLD. This case demonstrates the possibility of transporting both the mother and surviving infant A to a higher level of care prior to delivery of subsequent fetuses.

## 1. Background

The incidence of multiple-fetus pregnancies has dramatically increased over the past two decades due to assisted reproductive technology [1–3]. As a result, second-trimester preterm labor, PPROM, and fetal demise are more commonly encountered by perinatologists [1]. Despite advances in prenatal care, preterm delivery is associated with a high risk of neonatal mortality and morbidity [4]. Gestational age is the most important predictor of neonatal survival in extremely low birth weight babies [5]. In singleton, survival to discharge following delivery at 24, 25, and 26 weeks is 31.2%, 59.1%, and 75.3%, respectively [5]. In multiples, the mortality rate was 32% from 23 to 25 weeks' gestational age, compared to 19.2% from 26 to 27 weeks' gestational age and 11.1% in all gestational age [6]. At these extremely premature gestational ages, even small increases in gestational age have tremendous impact on neonatal survival [5]. Thus, a goal of pregnancy management is to prolong gestation and maximize fetal weight if a mother-fetus dyad is threatened with preterm delivery.

In a multiple-fetus pregnancy, the birth of the first fetus is usually followed by the delivery of the following fetuses. In some cases of multiple pregnancies, however, uterine contractions stop once the first fetus is delivered. The successful delay of the delivery of the second child can be life saving to the subsequent children. In particular, when a first twin is delivered prior to 24 weeks, delayed delivery of the second twin can be associated with reduced perinatal and infant mortality of the second twin [7].

U.S. Naval Hospital Okinawa, Japan, was scheduled to move 3 kilometers from its 55-year-old facility at Camp Lester, Okinawa, Japan, to a new state-of-the-art facility at Camp Foster, Okinawa, Japan, during the time of this case. In the literature, there is one reported case of delayed interval delivery involving transportation to another facility [8]. In this report, the first fetus was delivered at 17 weeks and was nonviable.

We report a case of a patient with a twin pregnancy with the survival of both twins following successful transportation of the mother and Twin A to another facility.

## 2. Case Presentation

Maternal history is as follows: 30-year-old G_5_P_1031_ African American female with history significant for diamniotic/dichorionic twin pregnancy with concordant growth presented to U.S. Naval Hospital Okinawa, Japan, at 22 + 5 weeks with preterm labor and advanced cervical dilation. At 23 + 2 weeks, she experienced PPROM and vaginally delivered Twin A. Following delivery of Twin A, Placenta A was left* in utero* with high ligation of the umbilical cord. Ultrasound demonstrated a reconstituted cervix and a long cervical length. There was no evidence of chorioamnionitis. The mother was administered 7 days of antibiotics to include 3 days of clindamycin and gentamycin and 4 days of oral cephalexin and metronidazole. No tocolytic medications were administered and no cervical cerclage was placed. Due to the scheduled hospital move, she was transported by ground ambulance to the new facility and discharged home after 3 days. She was readmitted at 26 + 3 weeks in active labor. During triage, Twin B demonstrated tachycardia and deep variable decelerations. Twin B was delivered vaginally within one hour of admission with two coordinated pushes. On pathology, Placentas A and B were fused and Placenta B was noted to have a large clot covering 50% of its surface.

Twin A history is as follows: Twin A was delivered at 23 + 2 weeks and was given positive pressure ventilation and surfactant in the delivery room. Her birth weight was 650 g (86th%) with APGAR scores of 6 (1 min) and 7 (5 min). On day of life (DOL) 5, Twin A was transitioned from conventional ventilation to HFOV for worsening respiratory status. On DOL6, dopamine was started for hypotension. The patient was unable to be transported to the new hospital in conjunction with the scheduled NICU move (planned for DOL12) due to worsening clinical status. She stabilized and on DOL16 was transported to the new facility without complications. Her hospital course was significant for no IVH, PVL, ROP, or BPD. She was discharged at 38 + 4 weeks' corrected age with a discharge weight of 2612 g.

Twin B history is as follows: Twin B was delivered at 26 + 4 weeks and was given positive pressure ventilation and surfactant in the delivery room. Her birth weight was 930 g (67th%) with APGAR scores of 3 (1 min) and 7 (5 min). Her hospital course was significant for no IVH, PVL, ROP, or BPD. Twin B was discharged at 35 + 6 weeks' corrected age with a discharge weight of 2666 g.

Hospital move was as follows: The U.S. Naval Hospital Okinawa was scheduled to move 3 kilometers from Camp Lester to Camp Foster. Nine days prior to the scheduled move, Twin A was delivered. Immediately prior to the move, Twin A's condition became more critical. The hospital leadership recognized the high risk of an unstable ELBW transport and chose to keep the Camp Lester NICU open until Twin A was more stable. Concurrently, the new NICU at Camp Foster was opened to provide support to the Labor and Delivery service. For four days the NICU, pharmacy, radiology, laboratory, and respiratory therapy departments provided 24-hour staffing at each location.

## 3. Discussion

Delayed interval delivery was first reported in the mid-20th century as a means to prolong pregnancy for multifetal gestations after the spontaneous second-trimester delivery of the first fetus. In 1957, Abrams published the first reported case of delayed interval delivery in a patient with normally shaped uterus [9]. Since then, several case reports and case series have been published [10–13]. Although these have clearly demonstrated that delayed interval delivery can be successfully achieved, no standard protocol for management exists because of its very rare occurrence. The use of prolonged bed rest, cervical cerclage, tocolysis, antibiotics, and corticosteroids composes complex, frequently debated issues [14]. In all cases, the umbilical cord of the first-born twin is cut as high as possible inside the cervix [15]. It is remarkable that the remaining placenta and umbilical cord of the expelled fetus do not seem to initiate intrauterine infection [16]. A known risk factor for delayed interval delivery failure is a previous cerclage. Patients with a previous cerclage during pregnancy are less likely to achieve significant latency intervals [17].

Infection is often implicated with preterm labor and preterm birth [18]. All possibilities of infections must be ruled out before attempting delayed interval delivery. In particular, clinical chorioamnionitis must be absent as this can lead to uterine contractions and subsequent delivery. In these cases, broad-spectrum antibiotics are often used to protect against ascending infection [14]. There is controversy about the administration of prophylactic antibiotics. Some authors suggest that antibiotics are not useful in this situation because many low birth weight deliveries occur without placental or amniotic fluid infection [19, 20]. On the other hand, Arias observed that the main reason for failure of delayed interval delivery was intra-amniotic infection [21]. Moreover, as antibiotics often have tocolytic properties, the use of broad-spectrum antibiotics seems justified [21]. In this case, antibiotics were used to mitigate the increased risk of infection associated with PPROM. Because the mother's uterine contractions had quiesced and a long cervical length was noted after the delivery of the first fetus, a tocolytic was not administered and a cervical cerclage was not placed.

Most studies demonstrate that maternal morbidities associated with delayed interval delivery are rare. However, Roman found a 31.6% incidence of serious maternal morbidity related to the delayed interval delivery [22]. All cases were associated with evidence of infection as demonstrated by either clinical signs, positive cultures, or placental pathology. However, most women who had serious morbidity had a negative amniocentesis for subclinical chorioamnionitis prior to undergoing delayed interval delivery [22]. Thus, the risk of a serious, potentially life-threatening maternal complication is difficult to predict and patients must be informed of both fetal and maternal risk during informed consent. In this case, amniocentesis was not performed after the delivery of first fetus.

Delaying the delivery of the second infant has a positive effect on the short-term outcome of that infant [23]. Long-term outcome is comparable to children with the same gestational age [23]. Delayed interval delivery may be attempted when the first baby is born before 24th week of gestational age in order to prolong the second infant's delivery until the 28th to 32nd week. This case is only the second report of delayed interval delivery in which neither twin suffered any major neonatal morbidity (no ROP, IVH, PVL, or BPD). Furthermore, this is the only case reported in which the first infant and mother survived without major morbidity after being transported to another hospital [7].

Interfacility transport of sick and premature infants is a common method to ensure that infants are treated at centers with optimum support. Newborn outcomes are improved if women are transported antenatally, especially for those preterm infants born at less than 30 weeks' gestation [24]. In delayed interval delivery, if infant A is delivered at a location without access to tertiary care, it is possible to transfer the mother to a more appropriate center prior to delivery of subsequent fetuses. While the purpose of this case's transport was to move to a new hospital, it demonstrated the ability to move both infant A and the mother to a center with an increased level of care.

In conclusion, this case demonstrates the possibility of transporting both the mother and surviving infant A without any complications to a higher level of care prior to delivery of subsequent fetuses, using delayed interval delivery.
